# P-634. Effect of Pre-existing OPV-induced immunity on development of canonical and non-canonical poliovirus virulence mutations

**DOI:** 10.1093/ofid/ofae631.831

**Published:** 2025-01-29

**Authors:** Yuan J Carrington, Frank Zhou, Jonathan Altamirano, Yvonne A Maldonado

**Affiliations:** Stanford University School of Medicine, Stanford, California; Stanford University School of Medicine, Stanford, California; Stanford University, Stanford, California; Stanford University, Stanford, California

## Abstract

**Background:**

Preventing vaccine-derived polio is crucial, yet polio evolution and transmission mechanisms remain unclear. Our previous study in Mayan infants found that preexisting OPV-induced polio antibodies did not affect S3 immunogenicity. We now explore if viral mutations differ in those with and without preexisting antibodies. Using NGS, we identify and characterize vaccine poliovirus’ mutation patterns from stool sample isolates post one and two OPV doses. We hypothesize that OPV-induced intestinal immunity will correlate with increased mutations after the 2^nd^ dose compared to the 1^st^.

S1 Canonical Mutations vs. Mutation Rates Post 1st and 2nd Dose of 116 OPV Isolates

**Figure:**
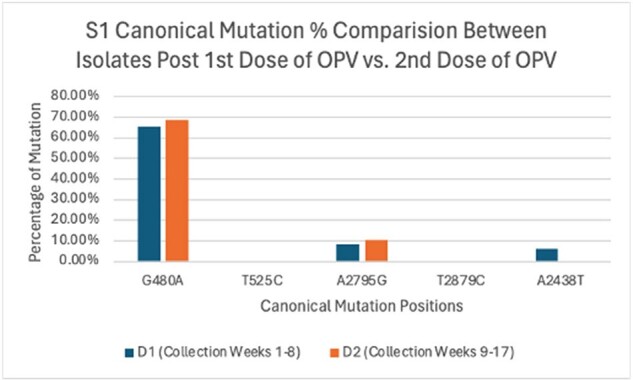

**Methods:**

179 OPV samples from a previous study were used. Participants received an OPV dose at study weeks 1 and 9 with stool samples collected at weeks 0, 1, 2, 4, 6, 8, 9, 10, 14, 16, and 17. A multiplex qPCR was used to determine serotype positivity. PCR amplicons created from stool viral RNA were purified and sequenced. Sequences were analyzed using the Illumina workflow nf-core/viralrecon (ver 2.5) and R (ver. 4.2.2).

S2 Canonical Mutations vs. Mutation Rates Post 1st and 2nd Dose of 82 OPV Isolates

**Figure:**
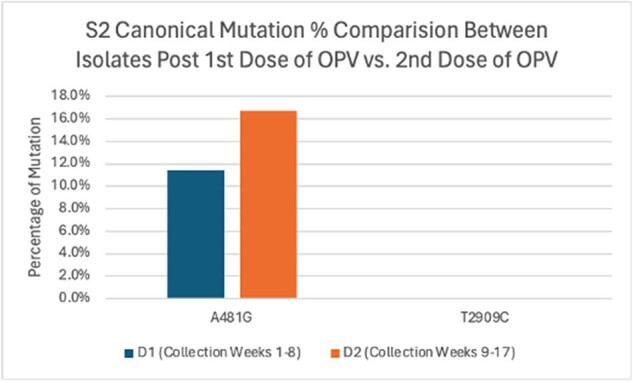

**Results:**

303 isolates were sequenced: 127 serotype 1 (S1), 91 serotype 2 (S2) and 85 serotype 3 (S3); 35 from week 0, 187 from weeks 1-8 (post dose 1: D1) and 81 from weeks 9-17 (post dose 2: D2). S1 and S2 in D2 showed significantly higher mutation rates (% of samples with mutation(s) at a given genome location) at all positions (S1 p = 0.0014, S2 p < 2.2e-16), while S3 showed no difference (p = 0.9899). For canonical mutations, S1 D2 had higher mutations at G480A (D1 65.3% vs. D2 68.6%) and A2795G (D1 8.5% vs. D2 10.5%), but 0% at A2438T while D1 had 6%; no mutations were seen at T525C and T2879C. For S2, D2 had higher mutations at A481G (D1 11.4% vs. D2 16.7%), with no mutations at T2909C in either set. In S3, D2 showed higher mutations at T472C (D1 36.6% vs. D2 46.4%), but lower mutations at C2493T (D1 26.9% vs. D2 16.7%) and T2034C (D1 3.8% vs. D2 0%).

S3 Canonical Mutations vs. Mutation Rates Post 1st and 2nd Dose of 70 OPV Isolates

**Figure:**
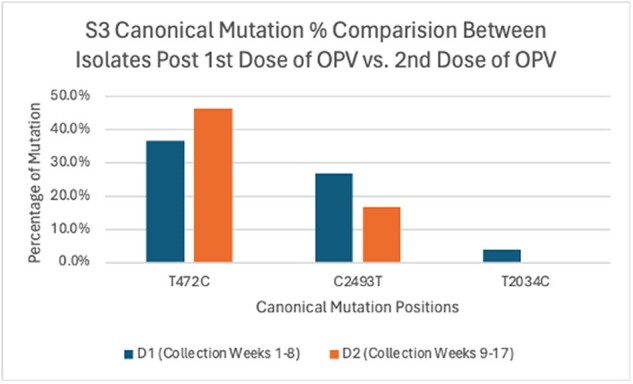

**Conclusion:**

Overall, isolates post the 2^nd^ OPV dose showed significantly higher mutation rates for S1 and S2, but not for S3. Preexisting antibodies may be associated with higher mutation rates and long-term likelihood of neurovirulent reversion. Serotype differences may be related to shedding patterns and other serotype specific characteristics. Further studies are planned to understand the impact of pre-existing intestinal immunity on polio viral mutation and evolution.

**Disclosures:**

**Yvonne A. Maldonado, MD**, Pfizer: Grant/Research Support|Pfizer: Member, DSMB

